# Eggshell ultrastructure and delivery of pharmacological inhibitors to the early embryo of *R*. *prolixus* by ethanol permeabilization of the extraembryonic layers

**DOI:** 10.1371/journal.pone.0185770

**Published:** 2017-09-29

**Authors:** Larissa Bomfim, Priscila Vieira, Ariene Fonseca, Isabela Ramos

**Affiliations:** Laboratório de bioquímica de insetos, Universidade Federal do Rio de Janeiro Instituto de Bioquímica Médica Leopoldo de Meis, Rio de Janeiro, Brazil; Fundacao Oswaldo Cruz, BRAZIL

## Abstract

Most vectors of arthropod-borne diseases produce large eggs with hard and opaque eggshells. In several species, it is still not possible to induce molecular perturbations to the embryo by delivery of molecules using microinjections or eggshell permeabilization without losing embryo viability, which impairs basic studies regarding development and population control. Here we tested the properties and permeability of the eggshell of *R*. *prolixus*, a Chagas disease vector, with the aim to deliver pharmacological inhibitors to the egg cytoplasm and allow controlled molecular changes to the embryo. Using field emission scanning and transmission electron microscopy we found that *R*. *prolixus* egg is coated by three main layers: exochorion, vitelline layer and the plasma membrane, and that the pores that allow gas exchange (aeropiles) have an average diameter of 10 μm and are found in the rim of the operculum at the anterior pole of the egg. We tested if different solvents could permeate through the aeropiles and reach the egg cytoplasm/embryo and found that immersions of the eggs in ethanol lead to its prompt penetration through the aeropiles. A single five minute-immersion of the eggs/embryos in pharmacological inhibitors, such as azide, cyanide and cycloheximide, solubilized in ethanol resulted in impairment of embryogenesis in a dose dependent manner and DAPI-ethanol solutions were also able to label the embryo cells, showing that ethanol penetration was able to deliver those molecules to the embryo cells. Multiple immersions of the embryo in the same solutions increased the effect and tests using bafilomycin A1 and Pepstatin A, known inhibitors of the yolk proteolysis, were also able to impair embryogenesis and the yolk protein degradation. Additionally, we found that ethanol pre-treatments of the egg make the aeropiles more permeable to aqueous solutions, so drugs diluted in water can be carried after the eggs are pre-treated with ethanol. Thus, we found that delivery of pharmacological inhibitors to the embryo of *R*. *prolixus* can be performed simply by submersing the fertilized eggs in ethanol with no need for additional methods such as microinjections or electroporation. We discuss the potential importance of this methodology to the study of this vector developmental biology and population control.

## Introduction

Fertilized eggs from oviparous animals are self-maintaining chambers that are able to fulfill the growing embryo with nutrients and energy required for cell growth, division and differentiation, so that development can be accomplished away from the maternal body. To generate a mature oocyte (i.e., an oocyte that is ready to be fertilized), the germline cells enter meiosis while they accumulate a massive storage of macromolecules such as proteins, lipids and carbohydrates (collective referred to as yolk), organelles and mRNAs. This accumulation generates a massive cell growth—up to 4.000x the original size—and a typically complex cytoplasm [1]. After growth, the last part of oogenesis is the synthesis of the chorion, or choriogenesis, where the multiple layers of the eggshell are synthesized and assembled, coating the whole surface area of the mature oocyte which is now ready to be fertilized and laid in the environment. In the species that colonized land, the chorion (or eggshell) is a further specialized protective shield for the embryos, being crucial to impair water loss and to allow gas exchange throughout development [2].

For most insects (including mosquito vectors of important arthropod-borne diseases such as malaria and dengue fever) silencing of target genes is usually accomplished by injecting dsRNA/siRNA in the thorax or feeding the adult animals, yielding a systemic silencing [3–6]. This type of approach is extremely useful for a variety of purposes, but it has the downside of resulting in phenotypes that can be difficult to interpret, since secondary effects are very common. The same rational can be used when pharmacological inhibitors are to be tested. The molecules are usually injected or fed to the adult animal, resulting in a hard to interpret-systemic effect.

Injecting vitellogenic females with pharmacological inhibitors and dsRNAs often results in the incorporation of the active molecules by the oocytes and inhibitor effect and gene silencing in the F1 progeny embryos. This type of effect has been extensively described and discussed in *R*. *prolixus* [7–13] as well as in other species [14–16]. Nevertheless, when a particular inhibitor effect or knockdown phenotype is detected at embryogenesis it is difficult to discern between an oogenesis-originated phenotype (since the mother was systemically affected) from phenotypes triggered at development. It is therefore critical to be able to deliver molecules directly to the embryo. Microinjections in the oocytes/embryos are commonly used in mainstream models like *C*. *elegans*, *Xenopus*, zebrafish and *Drosophila* [17–21], but most vectors of arthropod-borne diseases including mosquitoes, bugs and flies, produce larger eggs, with thick and rigid eggshells. For most of those species it is not possible or trivial to perform microinjections without losing embryo viability.

In 2005, the WHO created the department of the neglected tropical diseases (NTDs), recognizing their importance and aiming to manage their incidence mostly in Africa and Latin America (http://www.who.int/neglected_diseases/en/). The blood sucking bug *Rhodnius prolixus* is a strictly hematophagous vector of Chagas disease, one of the eight NTDs that are important in Brazil. Currently, 8 million people are estimated to be infected by Chagas disease, and vector control is still the most useful method to prevent this illness. It is acknowledged that the ability of insects to inhabit a variety of niches and become vectors of numerous diseases is partially due to their high reproductive outputs. Manipulations to interfere with the production/viability of the eggs/embryos are commonly used with the aim of population control. In this context, being able to manipulate and to induce molecular perturbations to the embryo is vital if one aims to reveal potential targets for interference in vectors reproduction. Rhodnius has been increasingly used for studies regarding insect immunity, vector capacity, and metabolism [22–24] and its whole genome sequencing was recently published [25].

The eggshell (chorion) is a specialized extracellular matrix that is secreted between the oocyte and overlaying somatic follicle cells during the later stages of oogenesis. It is a highly organized multilayered structure with regional specializations designed to perform a variety of functions. The composition and layers of the eggshell can vary enormously among different species, even within insects. Thus, delivery and permeabilization protocols are usually species-specific and experimental testing is necessary for non-mainstream models. Here, we describe the morphology and test the permeability properties of the eggshell of *R*. *prolixus*, with the anticipation to allow the delivery of pharmacological inhibitors to the embryo.

## Methods

### Chemicals

Colchicine, cycloheximide, digitonin, pepstatin and bafilomycin A1 were purchased from Sigma. Glutaraldehyde (grade I) was purchased from EMS.

### Insects

*R*. *prolixus* were reared in a colony maintained at 28°C and 70–80% relative humidity and 12:12 h light-dark cycle. The insects were fed with rabbit blood in an artificial apparatus according to Garcia et al. (1975) [26]. All animal care and experimental protocols were approved by guidelines of the institutional care and use committee (Committee for Evaluation of Animal Use for Research from the Federal University of Rio de Janeiro, CEUA-UFRJ #01200.001568/2013-87, order number 155/13).

### Scanning (SEM) and transmission (TEM) electron microscopy

Opercula from the eggs were carefully detached using a fine forceps and a razor blade. The samples were fixed for 4–6 hours in 2.5% glutaraldehyde (Grade I) and 4% freshly prepared formaldehyde in 0.1 M cacodylate buffer, pH 7.2. Samples were washed in cacodylate buffer, dehydrated in an ethanol series, critical point dried and coated with a thin layer of gold. Samples were observed in a FEI Quanta 250 scanning electron microscope. For TEM, samples were fixed and dehydrated as described above and embedded in a Polybed 812 resin. Thin sections were stained with lead citrate and uranyl acetate followed by observation in a Zeiss EM 900 transmission electron microscope, operating at 80 kV.

### Treatments with pharmacological inhibitors

Fertilized eggs were collected immediately after being laid or at further specific embryonic stages. The embryos were incubated for 5 min by immersion in 200 μl of the different solutions containing pharmacological inhibitors in a 96-well plate. Sodium Azide and KCN were incubated in ethanol 70%, bafilomycin A1 and pepstatin A were solubilized in ethanol 95%, because of poor solubility in pure ethanol. All other pharmacological inhibitors were diluted in ethanol >99.8%. For the experiments with ethanol pre-treatments, the eggs were immersed for 5 min in ethanol (>99.8%), allowed to dry for 1 min and then immersed for an additional 5 min in aqueous solutions of different inhibitors.

### Treatments with DAPI

Freshly laid eggs were immersed in DAPI 10 mg/ml solubilized in ethanol 50% for 5 min and 48h later the embryos were dissected and observed under a fluorescence stereomicroscope (Leica M165 FC). Alternatively, the eggs were immersed in ethanol (>99.8%), and then re-immersed in DAPI 10 mg/ml solubilized in water. 72h later, the embryos were dissected and observed under the stereomicroscope.

### SDS-PAGE

Bafilomycin A1 and Pepstatin-treated embryos were allowed to develop for 24h or 96h under the above specified conditions. Total protein homogenates were prepared in 10mM HEPES pH 7,2 supplemented with a protease inhibitors cocktail (aprotinin 0.3 μM, leupeptin 1μg/ml, pepstatin 1μg/ml, PMSF 0.1 mM and EDTA 1 mM). Forty μg of total protein were loaded in each lane and separated by a 7.5% SDS-PAGE. Gel was stained with silver nitrate [27].

## Results

### The eggshell allows solvent diffusion through the aeropiles in the operculum

**Fig 1:**
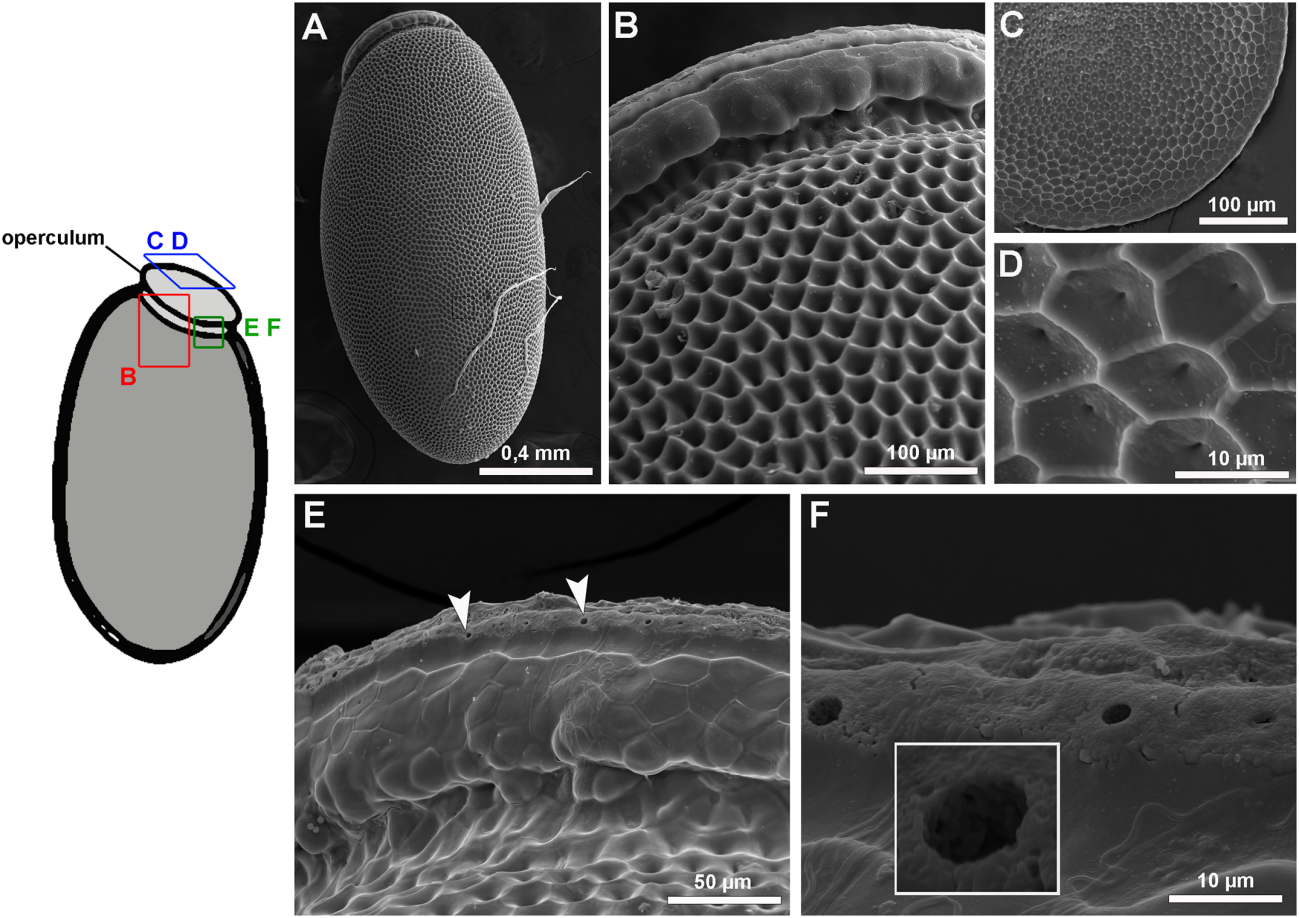
Aeropiles of the egg chorion in the operculum. Eggs were processed for field emission scanning electron microscopy and their surface ultrastructure was observed. Left, Sketch of the egg showing the operculum at the anterior pole and indicating the position of the images in the right panel. Right, (A) Low magnification micrograph of the egg. (B) Detail of a lateral view of the operculum as shown in red in the left. (C), (D): details of a frontal view of the operculum as indicated in blue in the left. (E), (F): High magnification images showing the aeropiles in the rim of the operculum (as indicated in green in the left). Inset in F, detail of an aeropile.

Freshly laid fertilized eggs were processed for FESEM and the ultrastructure of the external surface of the chorion was observed. The anterior pole of the eggs is closed by the operculum, which allows most of the O_2_ consumption during development and eclosion, as it functions as a lid that is pushed open by the nymphs for hatching [28] (Fig 1A and 1B). From a frontal view, the ultrastructure of the external surface of the operculum is marked by the presence of irregular pentagonal and hexagonal depressions, which are demarcated by the spaces where the follicle cells were placed at oogenesis (Fig 1C and 1D). Regularly spaced pores of 2 ± 0.2 μm in diameter were observed at the rim of the operculum (Fig 1E and 1F) as previously noted and observed by [29–31]. Presumably, most of the gas exchange between the embryo and the atmosphere is accomplished by those pores, named aeropiles. Aside from the operculum, the rest of the extension of the egg surface is covered by a stiff chorion with the external surface marked by the same pattern of depressions found in the operculum. No pores were observed under high resolution FESEM in any region of the eggshell apart from the operculum rim.

**Fig 2:**
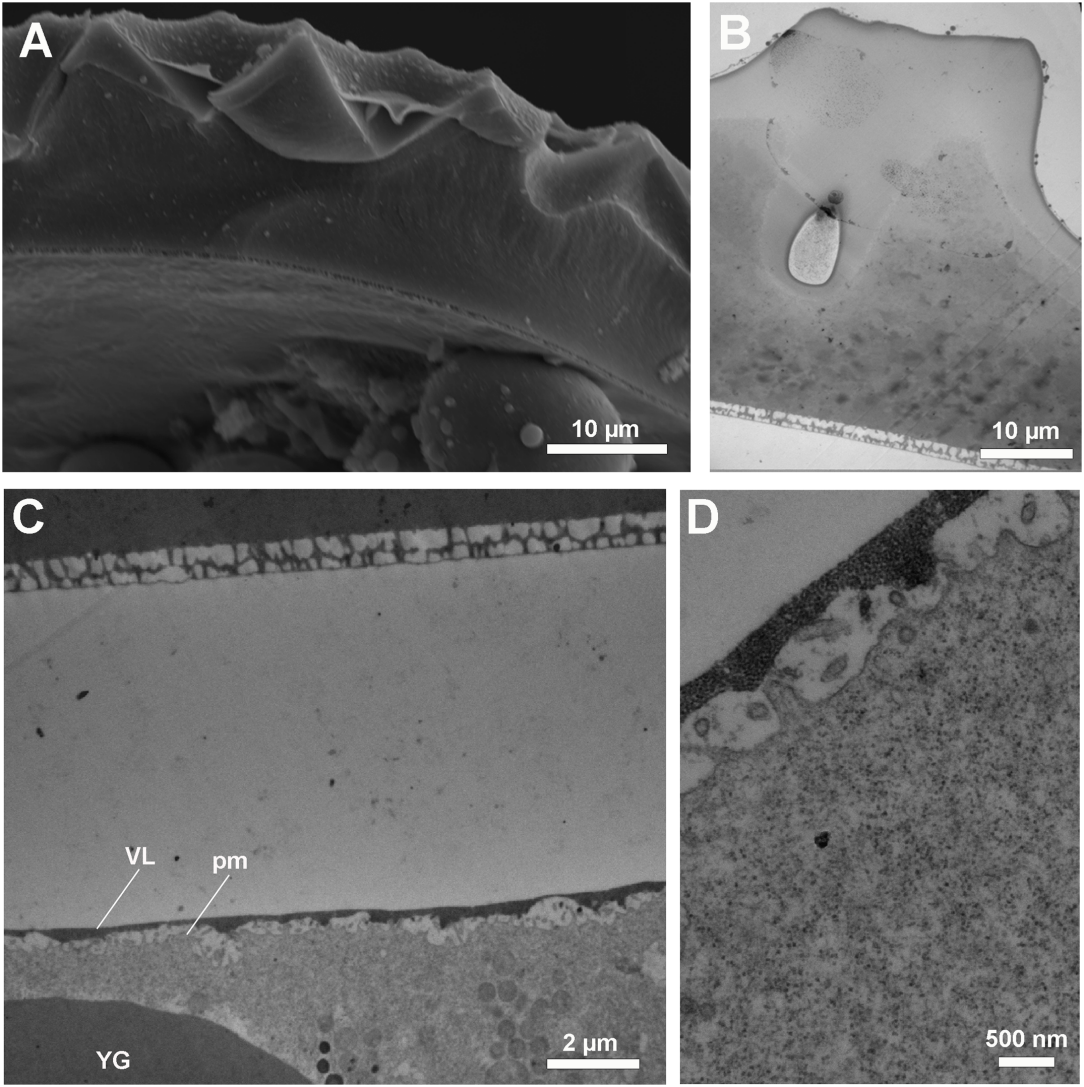
Transversal view of the egg chorion. Eggs were processed for transmission (TEM) and scanning (SEM) electron microscopy and their chorion ultrastructure was observed. (A) SEM image of a transversal section of the egg chorion. (B) Low magnification TEM micrograph of the egg chorion. Arrowhead: inner laeyr of the chorion. (C) Low magnification TEM micrograph of the egg peripheral cytoplasm showing part of the upper chorion (ch), the lower thin layer of the chorion (arrowhead), the vitelline layer (VL), the plasma membrane (pm), a large yolk granule (YG) and the peripheral cytoplasm (pc). (D) Detail of the vitelline layer and the plasma membrane.

**Fig 3:**
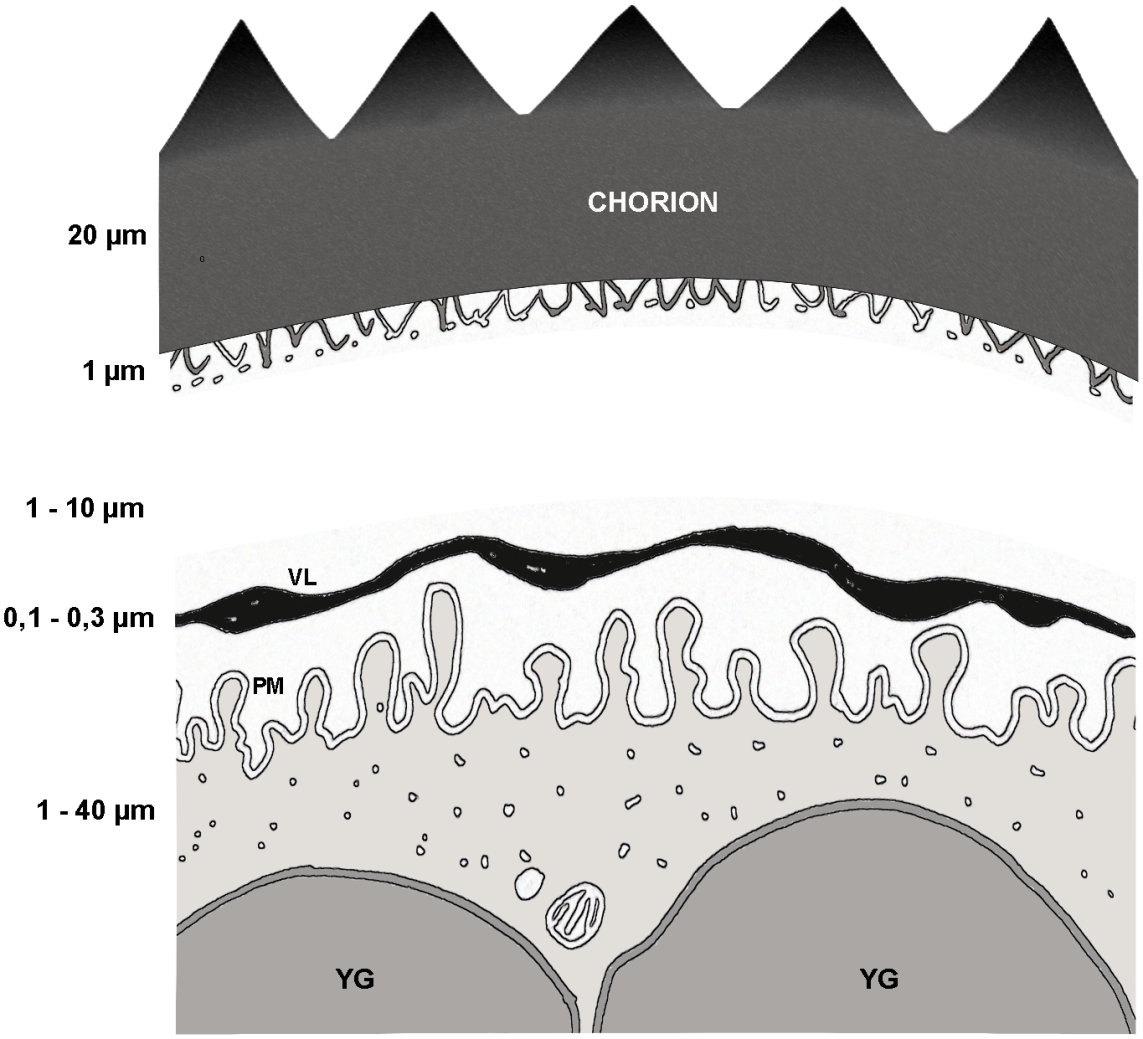
Illustration of the *R*. *prolixus* egg chorion and extraembryonic layers. VM, vitelline membrane (layer). PM, plasma membrane. YG, yolk granule.

To get a better assessment of the extraembryonic layers underlying the exochorion, transversal ultrathin (70–90 nm) sections of the eggshell were observed under the TEM. The chorion itself has two distinct layers. An inner thin layer (1 μm) formed by an uneven porous structure (arrowheads in Fig 2B), and an outer thick layer (20 μm) with uniform electron density (Fig 2B, Ch). Underneath the chorion there is a space between the thin layer and the vitelline layer (VL) (asterisk in Fig 2C). The vitelline layer has a higher electron density when compared to the chorion, and an irregular thickness that can vary from 1 to 3 μm. High magnification images, however, show that its structure appears to be porous (Fig 2C and 2D). Right below the vitelline layer we could see the egg plasma membrane (pm) enclosing the whole egg cell (Fig 2C and 2D). The egg extraembryonic layers of *R*. *prolixus* are represented in Fig 3.

**Fig 4:**
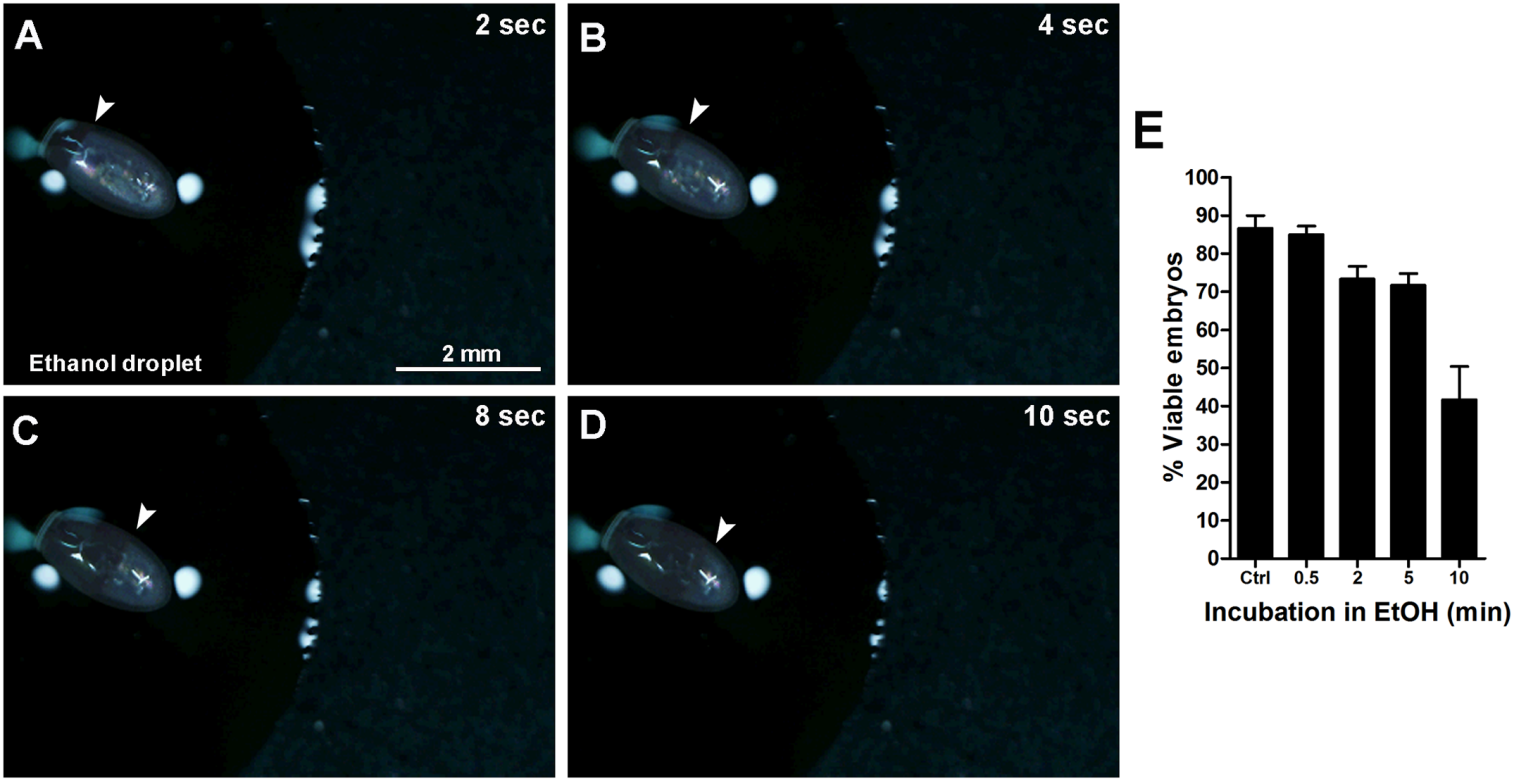
Time-lapse images of ethanol diffusion into the eggs. (A-D) Embryos were immersed in a droplet of ethanol and the entrance of the solvent was observed under the stereomicroscope. Arrowheads indicate the solvent diffusion front at the designated times in seconds. (E), embryo viability after increasing incubation times with ethanol. Graph shows mean ± SEM (n = 3).

To test if different solvents could reach the egg cytoplasm through the operculum pores, fertilized eggs (0-6h after egg laying, stage IA blastoderm embryos [7]) were immersed in a droplet of ethanol and observed under the stereomicroscope (Fig 4A–4D). Immersion of the egg in ethanol leads to a prompt entrance of the solvent through the operculum and rapid diffusion of the solvent across the anterior-posterior axis of the egg. It takes about 30 s for the solvent front to reach the posterior pole of the egg (Fig 4, arrowheads) (S1 Movie). The same test was performed with water droplets, and, visually, water does not appear to penetrate the chorion (data not shown). We further tested the viability of the embryos (successful hatching of the nymph) after ethanol diffusion, and found that eggs immersed in ethanol for 30 s show no differences in embryo viability when compared to control eggs, whereas 5 min ethanol immersions lead to a decrease in 20% in viability (Fig 4E). No differences in embryo viability were observed after water immersions up to 15 min (data not shown).

### Pharmacological inhibitors can reach the cytoplasm of the egg and disturb embryogenesis

**Fig 5:**
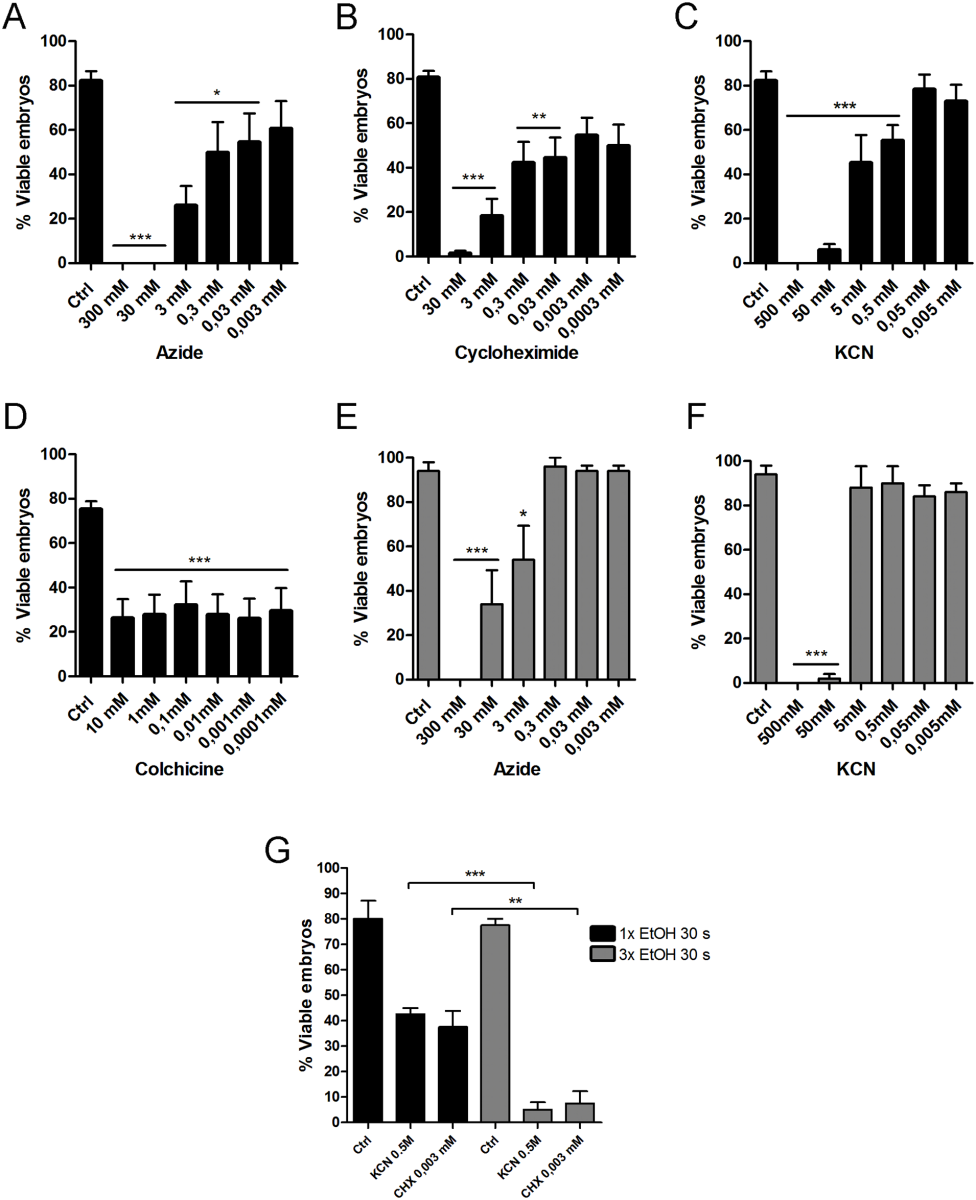
Embryo viability in eggs treated with pharmacological inhibitors. (A-D) 0-6h embryos were immersed for 5 min in solutions containing increasing concentrations of different pharmacological inhibitors diluted in ethanol (70% or >99.8%) (n = 10). (E-F) 96h embryos were immersed for 5 min in solutions containing increasing concentrations of azide or KCN diluted in ethanol (70% or >99.8%) (n = 10). (G) Embryos were immersed three times in KCN and cycloheximide solutions (n = 3). Graphs show mean ± SEM **p < 0.01, ***p < 0,001, one way ANOVA.

**Fig 6:**
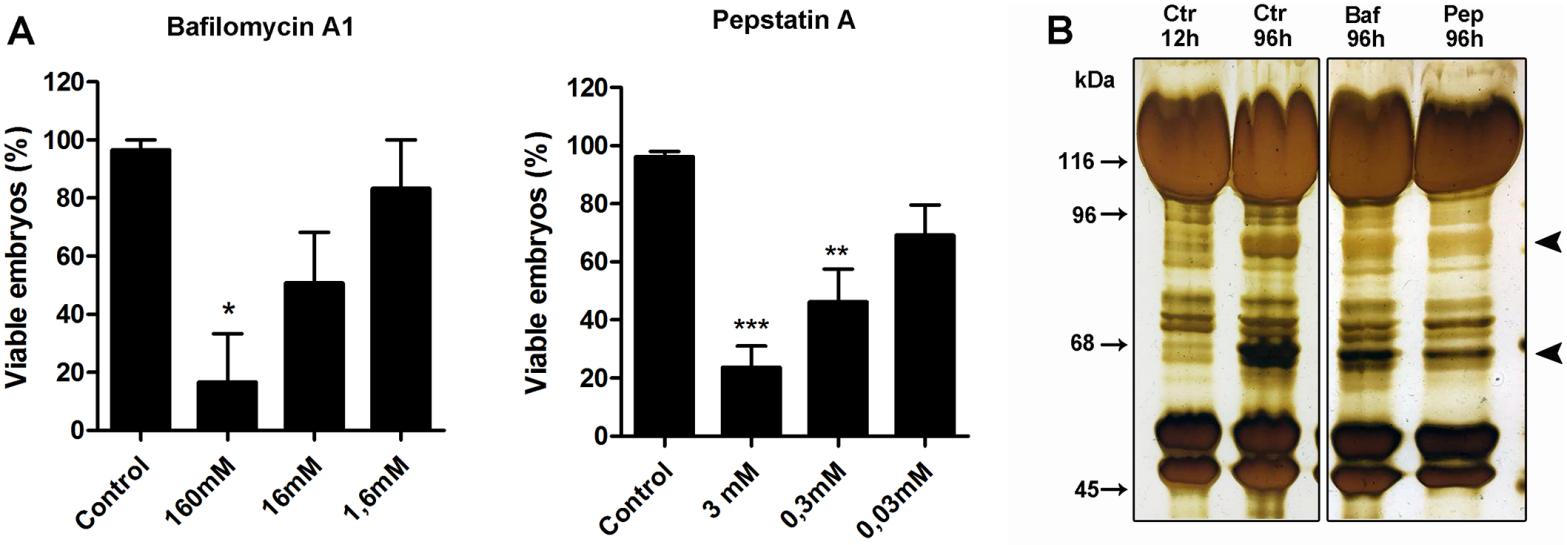
Embryo viability and effect of bafilomycin A1 and pepstatin-A on yolk proteolysis after delivery via ethanol permeation. (A) embryo viability after 5 min immersions in bafilomycin A-1 and pepstatin-A diluted in ethanol 95%. Graphs show mean ± SEM. Bafilomycin (n = 3), pepstatin (n = 5). *p < 0.05, one way ANOVA. (B) SDS-PAGE of 96h embryos after the different treatments showing the products of yolk proteolysis (arrowheads).

If one aims to deliver molecules to the cytoplasm of freshly laid eggs, assuming that ethanol can permeate and reach the space below the inner layer of the chorion, there still would be two barriers to transpose: the vitelline layer and the plasma membrane. Our next question was if ethanol itself could have a role in permeabilizing those layers/membranes. To test that, eggs (0-6h after egg laying) were incubated for 5 min in ethanol in the presence of increasing concentrations of different pharmacological inhibitors. Results show that sodium azide, potassium cyanide and cycloheximide were able to impair development in a dose-dependent manner when incubated with the eggs in ethanol, indicating that those molecules reached the egg cytoplasm and were delivered to the embryo (Fig 5A–5C). The embryos did not respond to colchicine (Fig 5D), an inhibitor of microtubular polymerization, in a dose-dependent manner, suggesting that the properties of the molecules (i.e. size, hydrophobicity, etc) influence on their penetrability through the extraembryonic layers. Additionally, we found that this delivery method is also effective to treat embryos later at development. Both KCN and azide treatments using eggs collected 4 days after egg laying (embryos at the onset of katatrepsis and dorsal closure [7]) resulted in impairment of embryo viability (Fig 5E and 5F). To test if we could increase the delivery efficiency of different drugs to them embryo, we tested if multiple immersions of the egg in the ethanol droplets would allow the entrance of more solvent, without decreasing embryo viability. Indeed, we found that multiple immersions (up to 3) did not affect embryo viability when compared to control groups. Also, immersing the eggs 3x for 5 min in KCN 0.5 mM in ethanol 70% further decreased embryo viability when compared to the 1x immersion experiment (Fig 5G). This indicates that the additional immersions resulted in the entrance of more solvent, allowing us to increase the final concentrations of the inhibitor reaching the egg cytoplasm. Our group focuses on the regulation of yolk degradation, so next we tested if known inhibitors of the yolk-targeting protease (an aspartyl protease—Pepstatin-A) and of the yolk organelles acidification (VH^+^-ATPase—Bafilomycin A-1) would have the expected effect—impairment of yolk degradation and embryo unviability—when delivered to the embryos through ethanol permeation. Results show that both pepstatin-A and bafilomycin A-1 resulted in a dose-dependent response in terms of embryogenesis viability (Fig 6A), and impairment of the yolk protein proteolysis, with pepstatin having a protective effect (Fig 6B). Overall, results show that different molecules can be delivered to *R*. *prolixus* embryos simply by incubating the egg for 5 min in ethanol, with no need of microinjections or additional permeabilization protocols.

**Fig 7:**
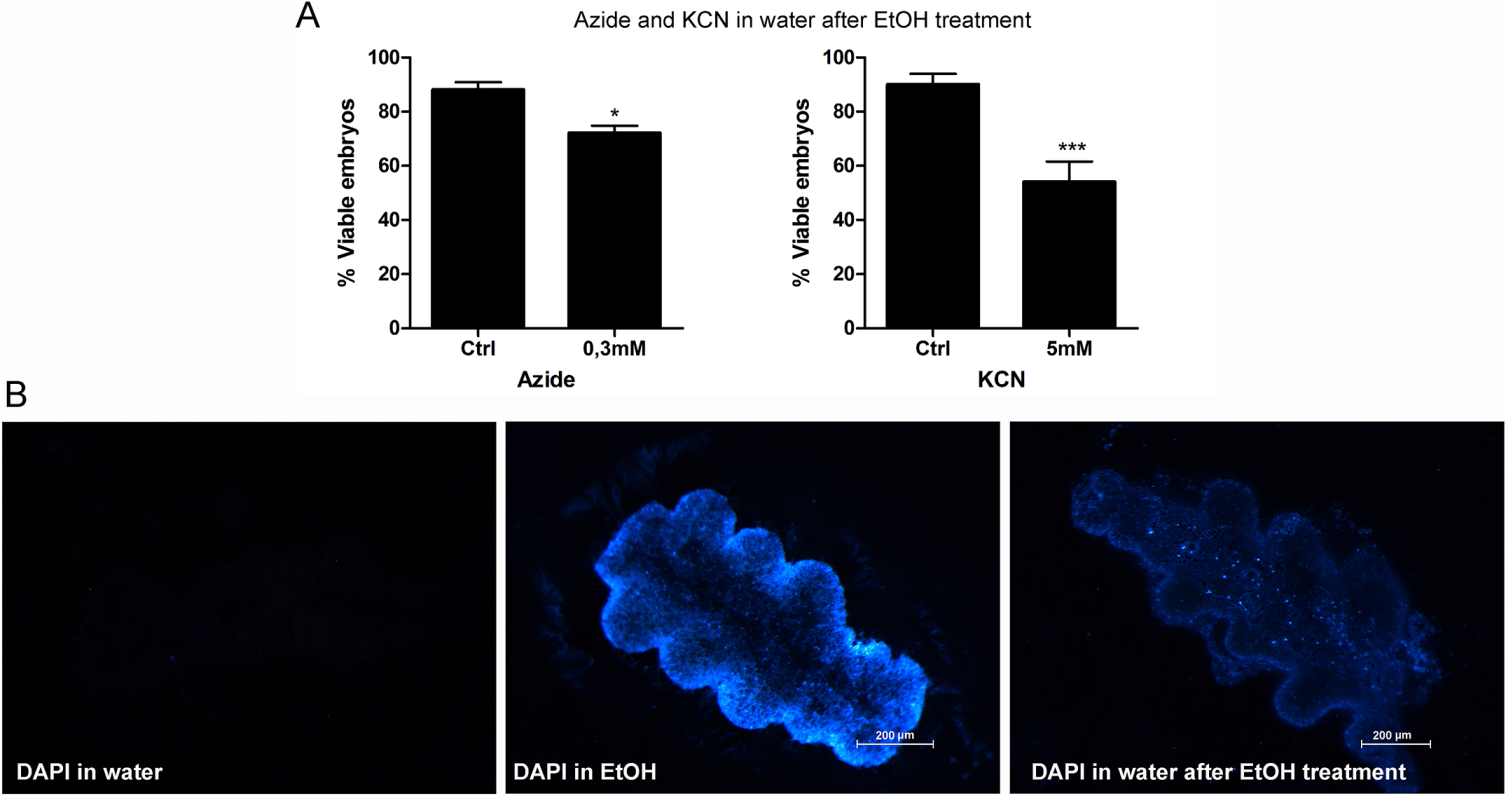
Aqueous solutions of azide, KCN and DAPI are delivered to the embryos when tested after the egg is treated with ethanol. (A) 0-6h embryos were immersed in ethanol for 5 min, allowed to dry, and then immersed in aqueous solution of azide or KCN. Embryo viabilities are shown (n = 8). Graphs show mean ± SEM. *p < 0.05, ***p < 0,001, one way ANOVA. (B) the same experiment was performed using DAPI at the concentration of 10 mg/ml (n = 5).

Beament (1945 and 1946) [32–34], showed that ethanol has an effect of wax removal from the inner layer of the chorion of Rhodnius eggs. To test if the effect of wax washout was sufficient to clear the way of the aeropiles and allow delivery of the pharmacological inhibitors in other solvents, we tested if aqueous solutions of azide and KCN were able to affect embryo viability when used after the egg was pre-treated with ethanol (presumably to wash off the wax the aeropiles). We found that, indeed, both azide and KCN had comparable effects in terms of embryo viability when solubilized in water after ethanol pre-treatment (Fig 7A) when compared to the experiments where molecules were directly solubilized in ethanol (Fig 5A and 5C). To further investigate this hypothesis, we made the same experiment using DAPI, which we anticipated would allow us to directly see the delivery to the embryo cells in the egg cytoplasm. As a result, we found that DAPI is able to reach the embryo cells equally, on both conditions (Fig 7B).

To test if we could further increase the permeability of the extraembryonic layers, we tested the embryos viability after immersions in increasing concentrations of different detergents, diluted in ethanol 50–70% or water. Digitonin, tween-20 and triton-X100 were tested, and concentrations up to 0.1% of all detergents did not affect embryo viability. Higher concentrations (1%), however, resulted in decreased viabilities of approximately 20%, 80% and 30% for digitonin, triton X-100 and tween-20, respectively, when compared to ethanol controls (S1A Fig). The non-toxic 0.1% concentration of all three detergents did not increase the delivery efficiency of KCN or cycloheximide to the embryos when solubilized in ethanol, indicating that detergents were not able to further permeabilize the extraembryonic layers (S1B Fig). We also made experiments adding detergents to aqueous solutions of KCN and cycloheximide, and found that it does not increase the effect of these drugs under these conditions (S1B Fig).

## Discussion

Like most insect eggs, regional specializations are apparent on the surface of *R*. *prolixus* eggshell. The operculum, at the anterior pole, has evolved to allow gas exchange throughout development and to facilitate hatching of the nymph at the end of embryogenesis. Using FESEM, we found that ethanol can diffuse through the aeropiles in the operculum and enter the chorion allowing the delivery of drugs and dsRNAs to the embryo. As a solvent, ethanol is moderately polar and has a lower surface tension when compared to water. These properties are likely the reason why this solvent better penetrates through the aeropiles.

Intracellular cleavages of *R*. *prolixus* embryos last up to 6 h after oviposition. Thus, if a molecule enters the egg cytoplasm any time before this point (blastoderm formation), it is likely that the forming embryo cells will uptake it, as long as it is dissolved in the cytoplasm. Depending on the molecule, our data shows that this can be achieved without any further manipulation of 0–6 h embryos after 5 min soaking in ethanol, as it is the case for KCN (65,12 g/mol), azide (65 g/mol) and cycloheximide (281,35 g/mol). For those drugs, multiple immersions were shown to increase embryo unviability, most likely because it allows a higher final concentration of the drug in the cytoplasm. Also, we found that 96 h embryos were also sensitive to azide and KCN, although slightly higher concentrations of those drugs were necessary for the same levels of embryo viability (when compared to the 0-6h embryos), it is clear that treatments with embryos later at development using this protocol are also effective. Plus, fenoxycarb (301,3 g/mol), which is usually used to mimic the effect of the juvenile hormone in insects, has been previously delivered to *Rhodnius* eggs using a topic application protocol with acetone as solvent. This treatment also resulted in unviable embryos in a dose dependent manner [35, 36]. Following the work of Beament, JW [33, 34, 37], which showed that ethanol can wash away the wax deposited in the endochorion of the aeropiles of *R*. *prolixus*, we found that after an ethanol pre-treatment of the egg, different drugs can be delivered to the embryos in aqueous solutions. Thus, it is likely that ethanol function is mainly to solubilize the wax and wash it away from the aeropiles, allowing other solutions to permeate through the channels and reach the cytoplasm. Thus, we describe here a simple methodology to deliver pharmacological inhibitors to embryos of *R*. *prolixus*.

It is important to emphasize that from all molecules that we tested colchicine (399,43 g/mol) was the only inhibitor that did not impair embryo viability as expected. The lack of a dose-dependent response of colchicine could be due to its physicochemical properties. Assuming that ethanol has in fact a role in washing away the wax so the liquid can penetrate the aeropiles (which is what our data and Beament’s work indicate, as discussed above) the drug solutions would reach the space between the vitelline layer and the chorion (see Fig 3). In this case, there still would be two barriers to transpose to reach the embryo cells in the cytoplasm: the vitelline membrane (VM) and the plasma membrane. The vitelline membrane is considerably thick, has an unknown composition, and we found it has a porous texture under the TEM. So, the diffusion through the VM is likely influenced by the drug physicochemical properties (for example molecular weight, molecular surface area, molecular volume, hydrophobicity). In one hypothesis, colchicine is the least polar of all the drugs tested in this work, this could be the reason of its poor diffusion through the VM, because of trapping by hydrophobic interactions. However, it is important to emphasize that since we do not know the exact composition of the extraembryonic layers, it is hard to get into final conclusions, but it is important to highlight that apparently not all drugs/ molecules can be delivered to embryos with this methodology.

Transgenic techniques are powerful methods for elucidating gene functions. However, such methods are available for only a few model organisms. Over the recent decades, because of its simplicity, RNA interference (RNAi) has been used as an alternative method for silencing specific genes, and it has enabled *in vivo* functional analyses of specific genes in non-model organisms. However, several technical obstacles persist, such as the low efficiency of the introducing dsRNA in some species [4]. With *R*. *prolixus*, it is easy to induce a systemic silencing by intrathoracic injections of dsRNAs in the adults, but delivery of macromolecules to the egg has proven challenging. Even in models that embryo microinjections are common and useful, it continues to be labor intensive and require individual manipulation of each embryo in a dedicated facility. Techniques that allow the delivery of macromolecules to a batch of embryos would be of great value and modified protocols that allow the delivery of dsRNAs and larger macromolecules to the embryo using ethanol as vehicle are currently under investigation in our laboratory.

## Supporting information

S1 Movie Embryo after immersion in a droplet of ethanol. The entrance of the solvent can be observed under the stereomicroscope.
(WMV)

S1 Fig Embryo viability in ethanol and aqueous solutions plus detergents. (A) Embryos were immersed for 5 min in solutions containing increasing concentrations of different detergents diluted in ethanol 50–70%. (B) Embryos were immersed for 5 min in ethanol or aqueous solutions containing 0.1% of the different detergents plus KCN or cycloheximide. Graphs show mean ± SEM (n = 3). **p < 0.01, ***p < 0,001, one way ANOVA.
(TIF)
